# Ethnobotanical profiles of wild edible plants recorded from Mongolia by Yunatov during 1940–1951

**DOI:** 10.1007/s40656-021-00428-0

**Published:** 2021-08-11

**Authors:** Yanying Zhang, Wurhan, Sachula, Yongmei, Khasbagan

**Affiliations:** 1 Institute for the History of Science and Technology, Inner Mongolia Normal University, Hohhot, 010022 People’s Republic of China; 2 College of Life Science and Technology, Inner Mongolia Normal University, Hohhot, 010022 People’s Republic of China; 3 Key Laboratory Breeding Base for Biodiversity Conservation and Sustainable Use of Colleges and Universities in Inner Mongolia Autonomous Region, Hohhot, People’s Republic of China

**Keywords:** Yunatov, The Mongolians in Mongolia, Wild edible plants, Ethnobotany

## Abstract

Mongolian traditional botanical knowledge has been rarely researched concerning the ethnobotany theory and methodology in the last six decades (Pei in Acta Botanica Yunnanica 135–144, 1988, as reported (Martin in Ethnobotany: A methods manual, Chapman and Hall, 1995)). However, most of the known literature of indigenous knowledge and information regarding the use of local wild plants among Mongolian herders was first documented by several botanical research of Russian researchers in Mongolia through the 1940s and 1950s. One of the most comprehensive works was completed by A. A. Yunatov (1909–1967), which is known as “*Fodder Plants of Pastures and Hayfields of the People*’*s Republic of Mongolia”* (FPM). Yunatov’s research sampled forage plants in Mongolia from 1940 to 1951 and subsequently published a study in 1954. The original transcript of FPM was later translated into Chinese and Mongolian (Cyrillic alphabet) during 1958 and 1968. In addition to morphological characteristics, distribution, habitat, phenology, palatability, and nutrition of forage plants, Yunatov`s record collected local names, the folk understanding and evaluation of the forage, as well as other relevant cultural meanings and the use of local wild plants (collected from the wild as opposed to cultivated plants) in FPM through interviews. The book contains the most precious records created in the 1940s and 1950s on folk knowledge of the Mongolians' wild plants in Mongolia. It was composed of 8 chapters and 351 pages in total. The fifth chapter of FPM, entitled “The systematic overview of forage plants,” making up 272 pages (77.49% of the total page counts). The order and content of the book-oriented along with profiles of specific plants. Yunatov collected detailed information on plants, such as the local name, morphology, distribution, habitats, ecological characteristics, and phenology. He also discussed the palatability of livestock, particular forage use, other usages, and chemical composition. Through careful reading and understanding of all three versions of the book (in Russian, Chinese, and Mongolian (Cyrillic alphabet)), the FPM-listed information of edible plants was categorized using ethnobotanical dependent analysis. The list of edible plants was ranked based on purposes and ethnobotanical inventories as per methodology and analysis used in the ethnobotan**y** research. FPM listed 35 species are part of 15 families and 25 genera of wild edible plants. Most species belong to Liliaceae and Allium. Naturally grown grain and some food substitutes (plants that could be used as substitutions for typical food) come from the starchy organs, such as seeds, bulbs, roots, and rhizomes of 12, accounting for 34.28% of all species. Wild vegetables come from the parts of a young plant, tender leaves, young fruits, lower leg of stems, and bulbs of 9 species, accounting for 25.71% of all species. There are only three species of wild fruits, accounting for 8.57% of all edible plant species. Tea substitutes consist of leaves, roots, follicle, and aboveground parts of 8 wild plant species, accounting for 22.85% of all species. Seasonings from the wild were made of the elements such as seeds, rhizomes, tender leaves of 7 species, accounting for 20.00% of all species (Fig,8). Similarities and differences are noticeable in utilizing wild edible plants among Mongolian populations living in Mongolia and Inner Mongolia. Six species of wild edible plants listed in FPM have been proven to be collected and consumed by Mongolians from the Genghis Khan era in the twelfth century to the present day. This proved that the Mongolians have a tradition of recognizing and utilizing wild plants, demonstrating historical and theoretical value. Seven species of plants mentioned in this book were closely correlated to the locals' processing of traditional dairy products, meat, and milk food. Yunatov was not an ethnobotanist, but his accurate documentation of interviews and surveys with Mongolians represents valuable information about the collection and consumption of local wild plants during 1940–1951 in Mongolia. His research mission meant to focus on forage grass, the feed plant that sustained livestock, while he also recorded plants consumed by humans. His records on the edible parts and intake methods of some plants are incomplete. Still, it provided ethnobotanical materials of a remarkable scientific value and a living history of ethnobotany in Mongolian regions. Even by today`s standards, it will be challenging to obtain first-hand information of the richness and to the extent of Yunatov’s research.

## Introduction

Currently, indigenous knowledge of wildlife and plants regarding their sources and usability in Mongolian culture is rapidly fading due to lesser economic significance in the current day modernization, continuing degradation of natural resources, and cultural homogenization of nomadic lifestyle to modern society. Wild edibles are no exception to this fact. Local knowledge of wild plants is vanishing along with the people who are knowledgeable of those, in the sense that it is slowly disappearing with the demise of those who have traditionally upheld it. Thus, it urgently requires thorough documentation of the traditional knowledge of plants and use. Ethnobotanists worldwide sensed the urgency to carry out ethnobotanical investigations and research about indigenous wild plants in Mongolian populated areas. The research interest in wild edible plants resurged in recent years, and the tradition of collecting wild edible plants has been preserved to date (Łuczaj et al., 2013; Lulekal et al., 2011). Studies have demonstrated that wild edible plants provide health benefits and therapeutic effects (Aryal et al., 2018; Jun Yang et al., 2020; Magsar et al., 2018; Ligaa et al., 2006; Zhiming et al., 2016). The use of wild edible plants represents a cultural and historical value and serves a sustainable organic lifestyle (Urtnasan Mandakh et al. 2020; Urgamal et al. 2019; Urgamal & Oyuntsetseg, 2017; Ju et al., 2013; Kang et al., 2014; Bhatia et al., 2018).

Mongolian Plateau is a suitable geographic area for ethnobotanical studies since there are still many Mongolian populations who attain indigenous knowledge and practice traditions about using wild plants. Ethnobotanical research about edible plants from the wild in the Mongolian Plateau was based on ethnobotany theory and methodology (Pei, 1988; Martin, 1995). Mongolians are a Central Asian ethnographic group comprising a closely related tribal with nomadic lifestyle populated across Mongolian Plateau and share a common language. Nowadays, the Mongolian population reaches approximately 10 million to date, and the majority are distributed among Mongolia, Inner Mongolia Autonomous Region. And there are some northwestern provinces and regions of China, such as Xinjiang, Qinghai Gansu China Kalmykia, and the Buryat Republics of the Russian Federation, have seen Mongolian ethnic populations. The traditional way of livelihood of the Mongolians has been nomadism. They mainly believe in Shamanism and Tibetan Buddhism and mostly wear Mongolian robes. Animal husbandry has a long tradition in Mongolia, and it is the foundation of the national economy and a primary resource of Mongolia’s food processing industry and daily necessities. Meat and milk are the main elements in the traditional diet of Mongolian herders. Plants, being the essential element in the daily portion, have exceptional functions and value among Mongolians who keep a nomadic lifestyle. Currently, the traditional botanical knowledge in Mongolia has rarely been studied, and most of these studies were based on the theory and methodology of ethnobotany in the past 60 years.

**Fig. 1:**
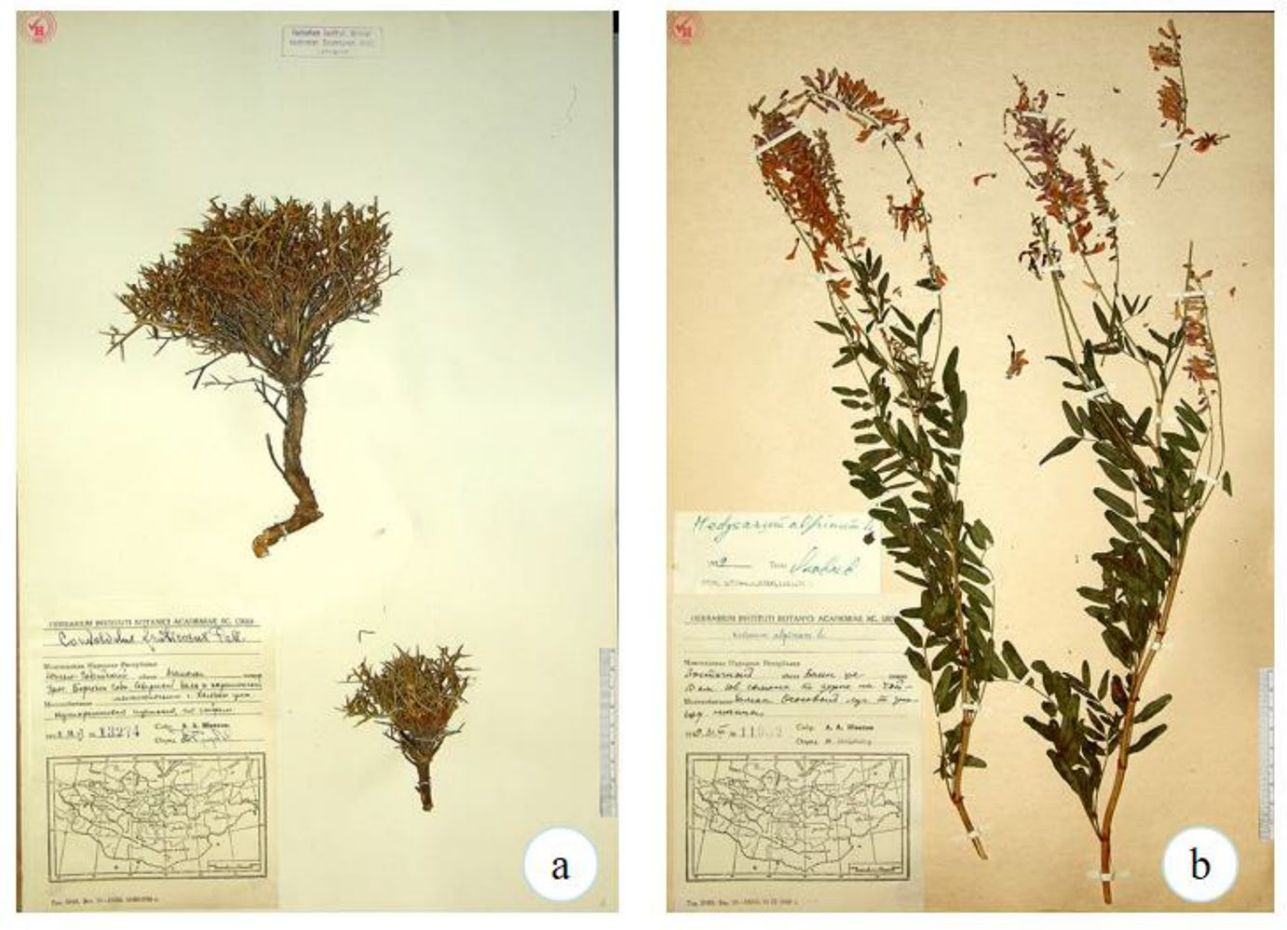
Specimens collected by Yunatov Source:(LE 2020)

However, a number of scholarly articles concerning indigenous knowledge of wild plants exist with some recorded valuable details of Mongolian herders using uncultivated plants. One of such existing reports were found in the literature of Russian scientists who studied Mongolian plants in the 1940s and 1950s. The most comprehensive work is known as "*Fodder Plants of Pastures and Hayfields of the People’s Republic of Mongolia*" (abbreviated FPM), completed by A.A.Yunatov. The book was published in Russian transcripts as Кopмoвыe pacтeния пacтбищ и ceнoкocoв Moнгoльcкoй Hapoднoй Pecпyблики (Yunatov, 1954). The author of the book―Yunatov, was a member of the Komarov Institute of Botany of the Soviet Academy of Sciences. He made frequent trips to Mongolia with the purpose of completing an agricultural research project. He served in the Scientific Committee of the People's Republic of Mongolia (PRM). During the field trips to the PRM, Yunatov organized several seminars on the experience of raising livestock with the participation of experts invited by the leader of the PRM (Horloogiyn Choybalsan). His great work was aided by his ability to speak the local language at a fluent level and his local assistant and translator (Trideep Olmde). At the time, all interviews were conducted with Yunatov himself, receiving assistance from experts of the Scientific Committee of the People's Republic of Mongolia and the Komarov Institute of Botany of the Soviet Academy of Sciences. Yunatov conducted a complete and systematic interview with each informant according to a specific outline. His sources included prominent livestock breeders familiar with local forage plants and foremost livestock experts at government-organized livestock experience seminars. The people chosen for this research were elders of all genders because of their broad collective knowledge of the wild edibles. Through this field research, Yunatov managed to collect more than 16,000 plant specimens, which had been preserved in the Vascular Herbarium of the Komarov Institute of Botany in Russia (Fig. 1) (LE 2020; Karamysheva, 2009; Volkov & Rachkovskaya, 2009).

Specimen a (*Convolvulus fruticosus* Pall.) original record: Specimens collected by Yunatov, Mongolian People's Republic, Southern Gobi *aimag*, Naipon somon, terrain feature Boruzon Gobi, northern bael [skirt] and adjacent minor hills of Khalzan Ula Mt., scrub desert, at sayrs, 18.VI.1949, coll. A.A.Junatov, No 13274; Representative specimen.

Specimen b (*Hedysarum alpinum* L.) original record: Specimens collected by Yunatov, Mongolian People's Republic, Eastern aimag, Bain Uul somon, 10 km south-east of somon, at the road to Choi-Balsan, sedge meadow on depression bottom, 31.VII.1949, coll. A.A.Junatov, No 11952; Representative specimen.

**Fig. 2:**
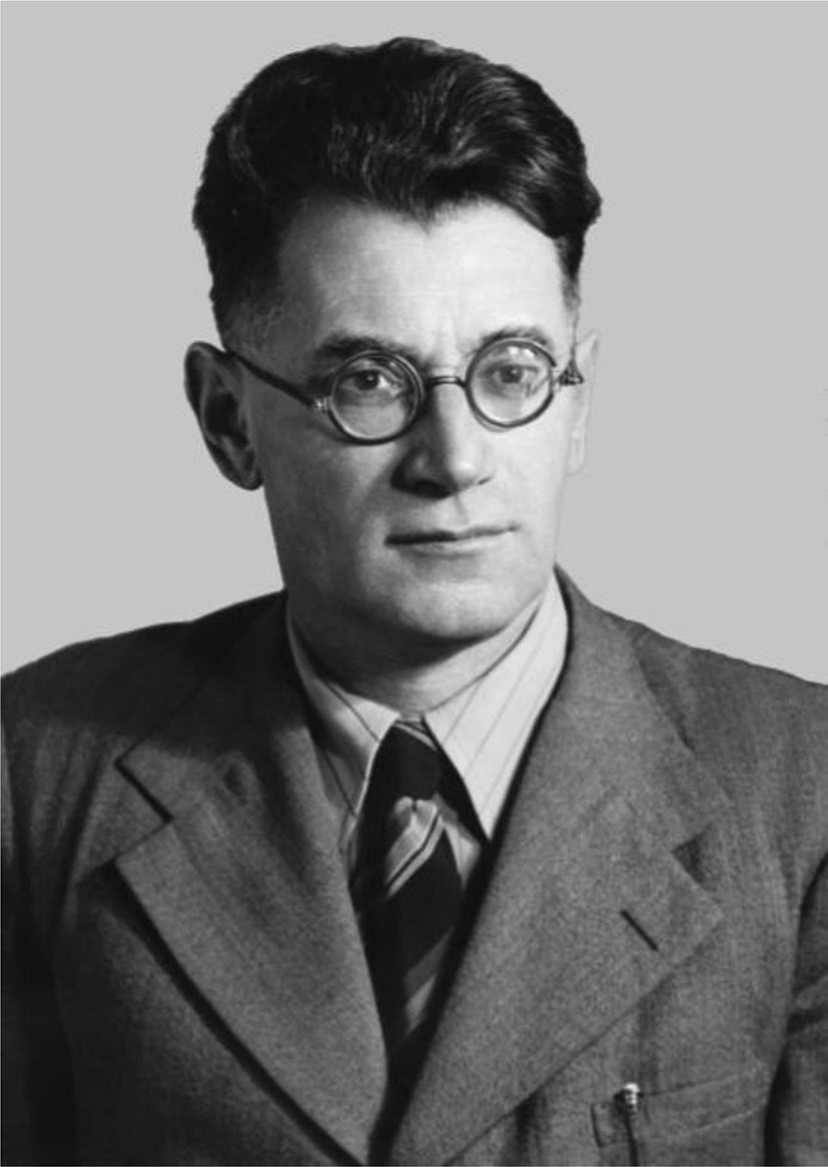
A. A. Yunatov (1910–1967), Source:(Z. V. Karamysheva, 2009)

**Fig. 3:**
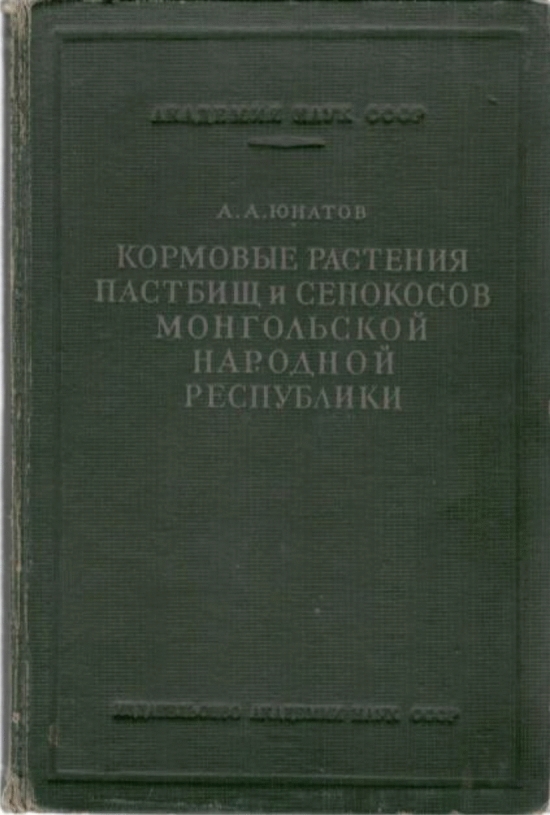
Original edition FPM, 1954

Alexander Afanasievich Yunatov (Aлeкcaндp Aфaнacьeвич Юнaтoв)(1909–1967) was a renowned botanist in the former Soviet Union, he was known for his excellency in geobotany, phytogeography, and plant research in Central Asia. Yunatov’s professional training started from enrollment at the Department of Biology, Leningrad National University, in 1935. The following year, he was transferred to Moscow State University, where he studied botany and graduated with excellence in 1940. Later, he received his associate doctor's degree in Life Science in 1948 and his doctor's degree in Life Science in 1954. He took the leading role in his discipline until he was promoted to Professorship in 1961 (Fig. 2)(Karamysheva, 2009). Yunatov proved himself to be a distinguished researcher and was awarded the Polaris Medal of the People's Republic of Mongolia in 1945 and honored the Komarov prize in 1951 for his remarkable service in science (Karamysheva, 2009; Volkov and Rachkovskaya, 2009). From 1940 to 1951, he participated in expeditions in Mongolia for research in forage plants. As one of his research results, he summrised all the field notes and composed FPM in 1954 (in Russian)(Fig. 3)(Yunatov, 1954).

**Fig. 4:**
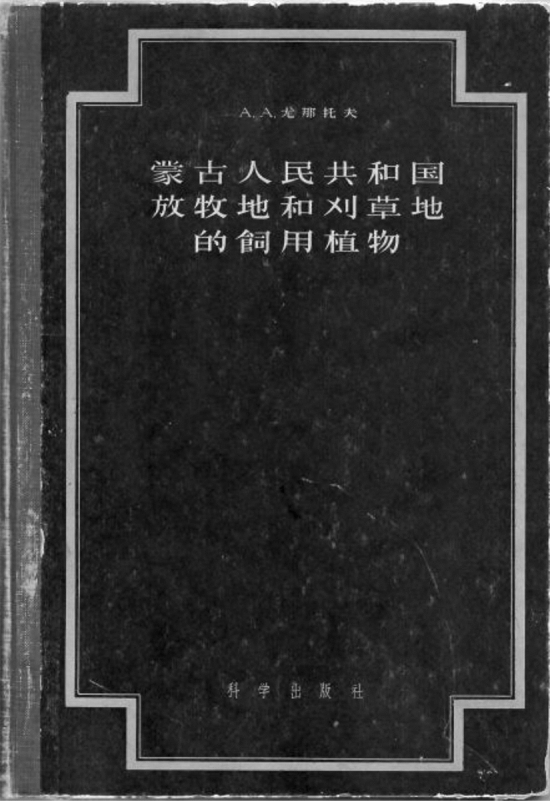
Chinese version of FPM, 1958

**Fig. 5:**
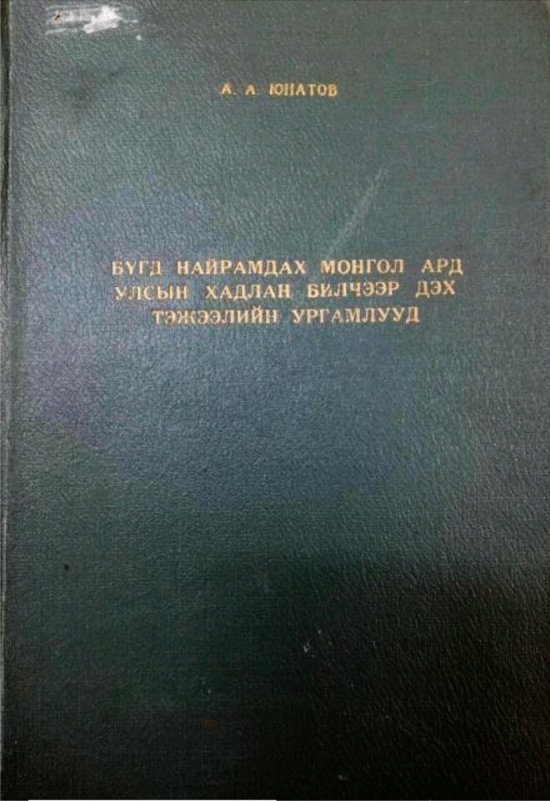
Cyrillic Mongolian version of FPM, 1968

Huang ZH, Ma YQ, and Wang JW translated FPM into Chinese in 1958. The Chinese edition was published by China's Science Press (Fig. 4)(Yunatov, 1958). Prof. G. Erdenjav brought FPM into Cyrillic Mongolian in 1968 (Fig. 5) (Yunatov et al., 1968).

Apart from FPM, Khasbagan et al. carried out some research since the 1990s about ethnobotany and history in Inner Mongolia, Khasbagan et al. studied transcripts of wild edible plants in the *Secret History of the Mongolians* (1240) (Khasbagan, 1996). and the ethnobotanical value of the book in terms of range management (Zhang & Khasbagan, 2016). A recently published article on textual research on the Mongolian names of Gramineous forage in the book "*Advice to the People on How to Manage Animal Husbandry*" belongs to ethnobotanical information the classical literature (Gilbaa & Khasbagan, 2019). Jamsrangiin Sambuu (1895–1972), who served as the Head of the People's Republic of Mongolia, compiled the book and summarized herdsmen's grazing experience in Mongolia (Sambo, 1945).

Therefore, it is necessary to arrange, inventory, analyze and evaluate the ethnobotanical information recorded in the book. The current research aimed to investigate ethnobotanical information of the published literature regarding wild plants among Mongolian ethnics. The present paper analyzed the information about the direct use of edible plants by herdsmen in Mongolia. The local names of plants and the knowledge of grazing use (indirect method) will be studied and written about separately.

## Materials

**Fig. 6:**
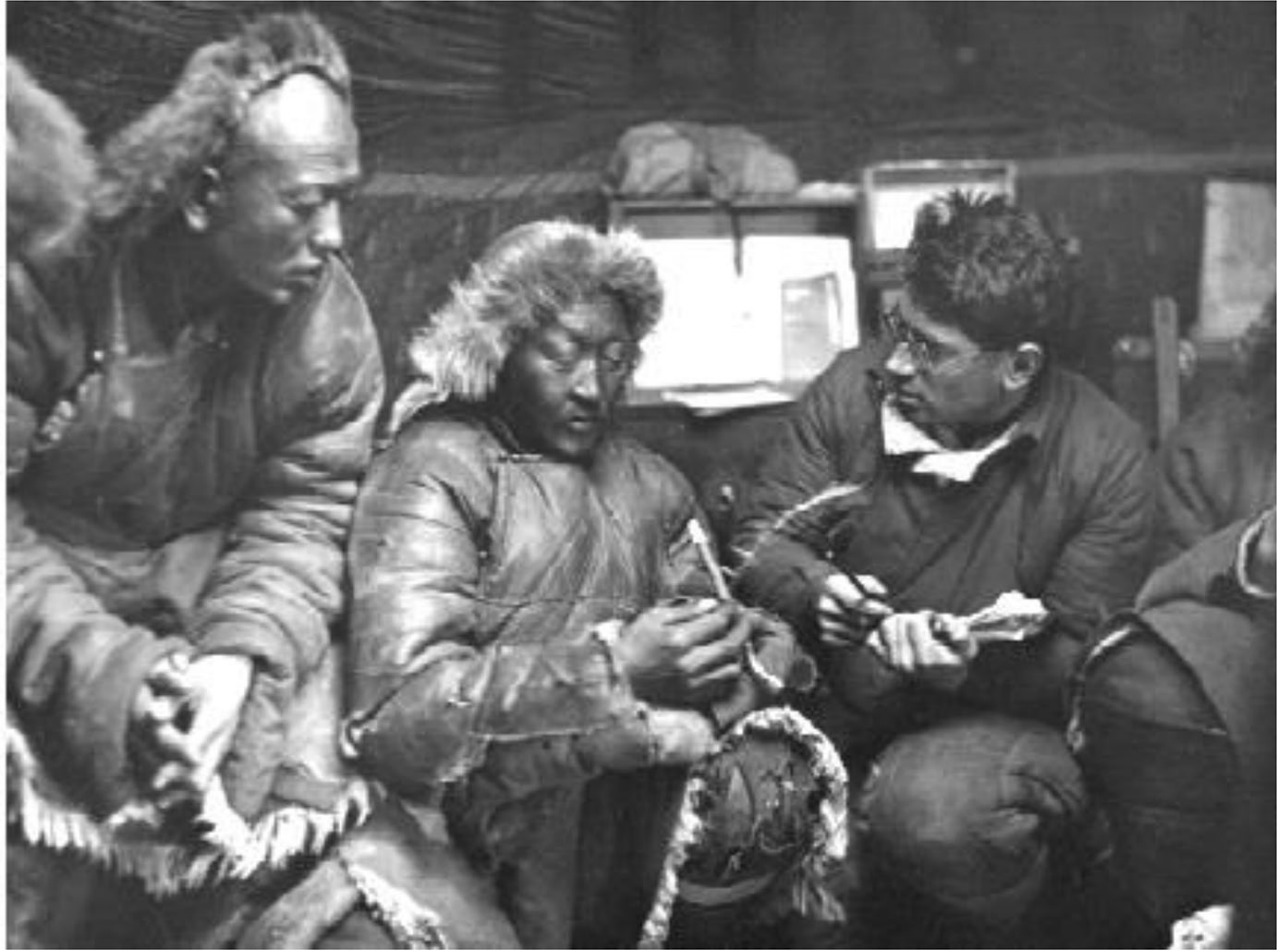
Yunatov was interviewing Mongolians, Source:(Z. V. Karamysheva, 2009)

**Fig. 7:**
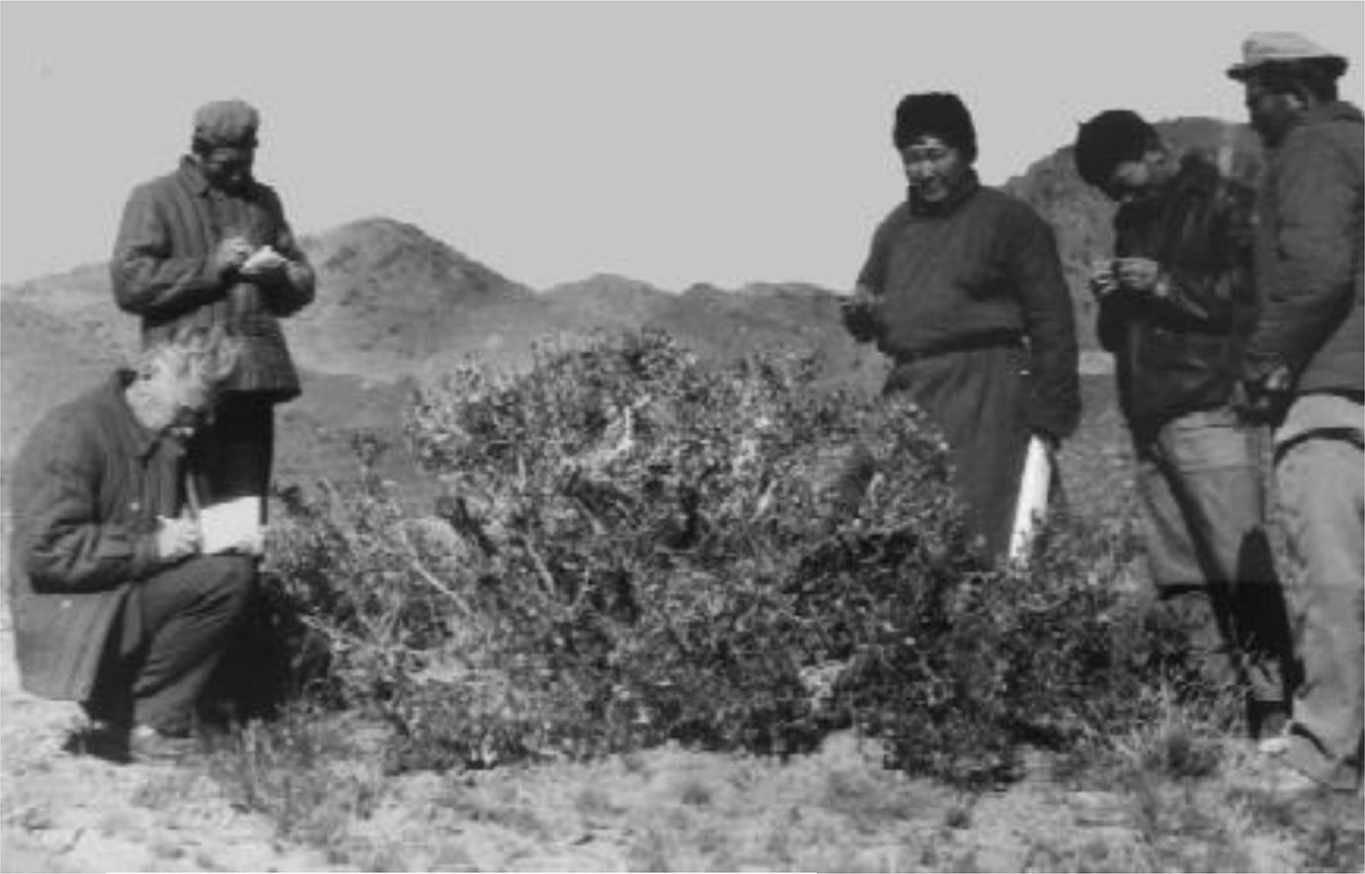
Yunatov and the Mongolians in the field, Source:(Z. V. Karamysheva, 2009)

**Fig. 8:**
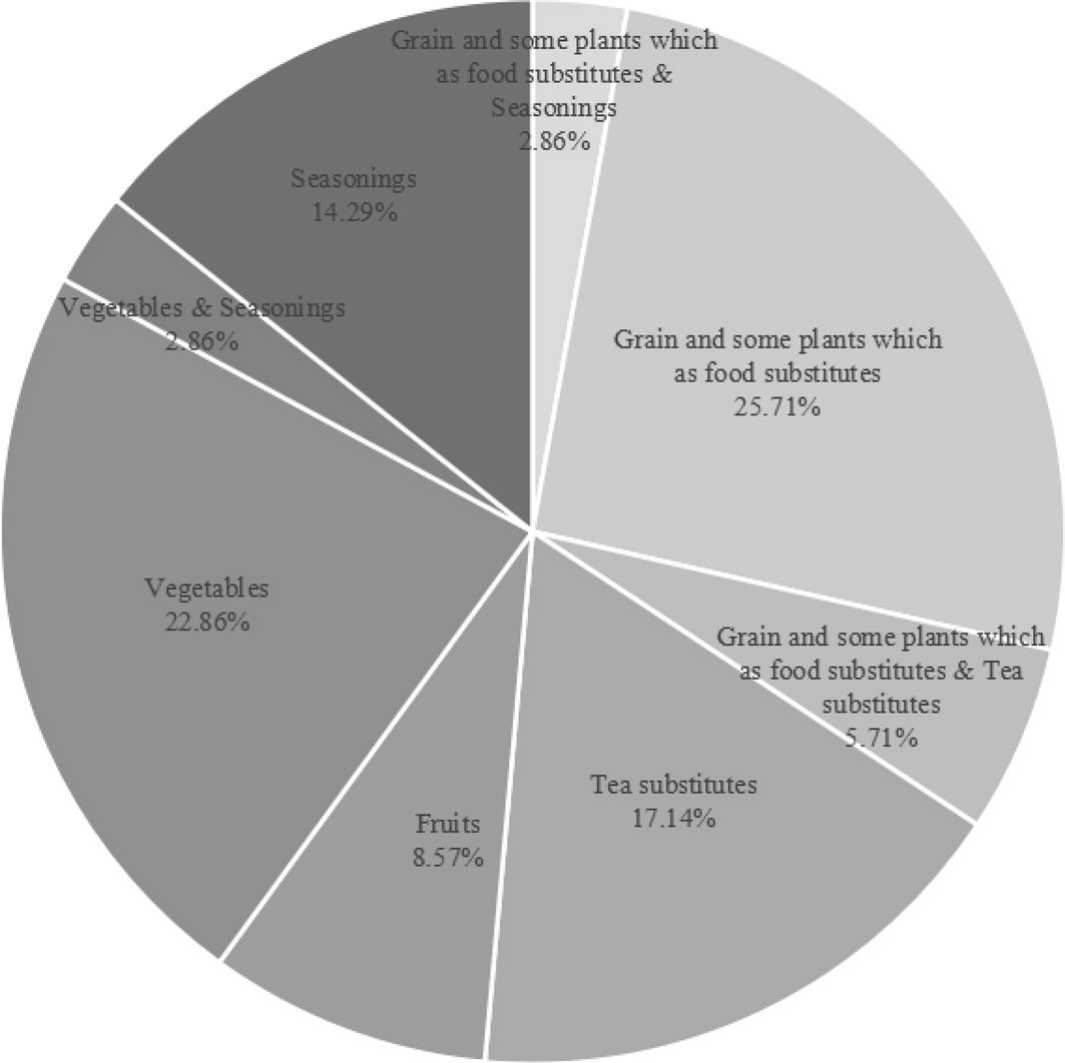
Wild edible plants and proportion of species arranged in food categories

In FPM, Yunatov recorded local names, folk understanding, and evaluation of the forage value and other relevant cultural meanings and uses of local plants. It included field interpretations of tremendous value following conventional records, such as morphological characteristics, distribution, habitat, phenology, palatability, and nutritional specification of forage plants. The author's view on folk plant knowledge is entirely consistent with ethnobotany theory from the book's introduction. The author's interviewing method in his fieldwork was precisely the same as the critical informant interview methodology (Figs. 6, 7, 8). In the analysis and evaluation of folk nomenclature and classification, the Russian adjective ethnobotany (Etnobotanicheskiy, in Russian) was used in a sense similar word of ethnobotany in English. Therefore, it could be inferred that the author possessed the knowledge and concept of ethnobotany while composing the book. According to the current data, this book has the most abundant folk knowledge of wild plants used by the Mongolian herdsman in Mongolia during the 1940s-1950s.

FPM was composed of 8 chapters and 351 pages in total. The first chapter was the preface, written by Lavrenko (1900–1987), a famous botanist in the former Soviet Union. The second chapter is an introduction, and the third chapter is about the “utilization of forage plants under the condition of Mongolian nomadism.” It described the ethnobotanical viewpoint of the author and the interviewing methods he used. The fourth chapter consists of “a brief introduction of various Pastures and Hayfields connected with national natural characteristics.” It mainly introduced the central vegetation zone and its related vegetation types of pastures and hayfields and natural regionalization of the people's Republic of Mongolia. The sixth to eighth chapters were the conclusion, references, and a list of forage plant names in Latin, Russian and Mongolian.

The fifth chapter of FPM was called “A systematic overview of forage plants,” and it comprises a crucial chapter of the book. The fifth chapter has 272 pages, accounting for 77.49% of the entire book. It elaborated on plant families, genera, and species in order of Pteridophyta (2 families, two genera, three species), Gymnospermae (2 families, two genera, five species), and Angiospermae (62 families,187 genera, 546 species). In the Angiosperm, Monocotyledoneae had been listed in the front of Dicotyledoneae. In Dicotyledoneae, the Salicaceae was arranged at first. Such ordering proved that the families of Angiosperm in FPM were set according to Engler and Parantl’s early classification system (Engler, 1930). The order and content of elaboration were specified to a specific plant, including profiles of local Mongolian names, morphology, distribution, habitats, ecological characteristics, phenology, palatability to livestock, particular forage use, usage for local people themselves, and chemical composition. Among them, local Mongol names collected by Yunatov were the original materials for studying Mongolians’ plant folk nomenclature and classification. The local knowledge of plant palatability to livestock contains what species or group of plants are suitable for which animal to feed. It contributes the raw material for studying the traditional knowledge of grazing plants of Mongol herdsmen.

The search of literature included lists of edible plants and distribution of given areas (Khasbagan et al., 2000, 2005, 2011; Wurchaih & Khasbagan, 2017; Man et al., 2007), wild edible fruits (Khasbagan et al., 1995; Soyolt et al., 1999; Enhebayar et al., 2002; Khasbagan et al., 2007), wild vegetables (Wujisguleng & Khasbagan, 2010; Sachula et al., 2020), tea substitute plants (Khasbagan, 1990; Khasbagan et al.^1996^), and ethnobotany of specific plant taxa related to food in some aspects (Khasbagan & Soyolt, 1996; Khasbagan et al., 1999; Khasbagan and Pei, 1999; Khasbagan & Soyolt, 2007) since the 1980s.

## Methods

### Data arrangement

The records of plants with local edible use in FPM were arranged through thorough reading and understanding among three versions of the book. This step is presumably ethnobotanical meta-analysis, and fortunately, it had been completed by Yunatov as early as 70 years ago.

### Revision of scientific names

Some of the author's scientific names in FPM were synonyms complied with the current taxonomy. According to the International Code of Botanical Nomenclature (ICBN), some plants’ popular names were found and adopted.

### Categorization and ethnobotanical inventories

The categorization of edible plants in FPM was based on purposes of use. Ethnobotanical catalogues were made following Pei SJ and Martin GJ. Guidelines (Pei, 1988; Martin, 1995). The tables are arranged alphabetically by the scientific name in all tables.

## Results

### Taxonomic features of wild edible plants

A total of 35 species of wild edible plants were recorded in FPM, which belong to 15 families and 25 genera. On the level of family, the report of ten species of Liliaceae was very prominent. Also, six species of Rosaceae, three species of Chenopodiaceae, two species of Poaceae, Polygonaceae, Ranunculaceae, and Brassicaceae were shown of the other eight families contained only one species. On the level of the genus, it has recorded eight species of *Allium* with prominence. Moreover, it has reported two species of *Lilium*, *Potentill*a, and *Paeonia*, and it showed each of the other 21 genera with only one species.

### Food categories

According to the original records, the author created five food use categories based on usage mode by the folk to classify wild edible plants. The groups included naturally grown grain and some plants. They are food substitutes, wild vegetables, wild fruits, tea substitutes, and seasonings sourced from wild (Table 1). *Allium lineare* L. was reported to be used as both vegetable and Seasonings, *Paeonia lactiflora* Pall. Furthermore, *Paeonia anomala* L. used both plants as food substitutes and tea substitutes, and *Polygonum viviparum* L. was recorded to use both plants as food substitutes and seasoning for culinary.

**Table 1:** Wild edible plants and number of species arranged in food categories

Food Categories	Grain and some plants which as food substitutes	Vegetables	Fruits	Tea Substitutes	Seasonings	Grain and some plants which as food substitutes & Seasonings	Grain and some plants which as food substitutes & Tea substitutes	Vegeta-bles & Seasoni-ngs
Species Number	9	8	3	6	5	1	2	1

### Original records, ethnobotanical inventory, and discussion

#### Wild Grain and some plants which as food substitutes

The original records of wild grain and some plants that serve as food substitutes in FPM were translated by the author of this article as follows:

- *Agriophyllum squarrose* (L.) Moq. [*Agriophyllum arenarium* M. B.](The author note: The scientific name in square brackets is the name adopted in the original text, and the accepted name is taken in this paper.): Since ancient times, the locals have collected a large number of seeds in a good year and made them into rice or flour for consumption.
- *Corispermum mongolicum* Iljin.: The locals collected its seeds, mashed them into powder, and fried them to process for food.
- *Kalidium gracile* Fenzl: Seeds were collected, ground into a powder then fried for meals.
- *Leymus racemosus* (Lam.) Tzvel. [*Elymus giganteus* Vahl]: It has seeds that are similar to *Psammochloa villosa*. Mongolians have a long tradition of collecting them since ancient times. And it is used as food in the form of grain and dried flour.
- *Lilium martagon* L.: Used in the form of fresh or dried bulbs for food by the locals.
- *Lilium pumilum* DC. [*Lilium tenuifolium* Fisch.]: Bulbs have been widely used as food. The newly harvested (usually in autumn) bulbs were often boiled in milk or consumed with buttermilk. Bulbs could also be purchased for storage in the rind (the floating film of butter) or oil. The locals considered this kind of oil or milk skin as good food.
- *Paeonia lactiflora* Pall.[*Paeonia albiflora* Pall.]: The local people cooked the fat and tuberous root as food or made into food similar to oat flour.
- *Paeonia anomala* L.: People used it in the same way as the previous species (note: the previous species in FPM was *Paeonia lactiflora*).
- *Polygonum viviparum* L.: Wild mice collect many starchy rhizomes for winter and the rations are hidden in special underground storage. Hence, the rhizomes of Polygonum viviparum were usually obtained from the underground warehouses of wild mice. Such practice had been performed since ancient times. The collected rhizomes of *Polygonum viviparum* would be cooked and applied as seasoning for cooking meat or flour.
- *Potentilla anserina* L.: The local people use starchy roots for food.
- *Psammochloa villosa* (Trin.) Bor: The locals usually harvest its caryopsis and use it for food in flour and rice.
- *Rheum nanum* Siev. ex Pall.: The roots were dried and ground into flour for making pancakes by herdsmen.

Wild grain and some plants as food substitutes are sourced from the starch-rich parts, such as seeds, bulbs, roots, and rhizomes, comprising 12 species of wild plants that account for 34.28% of all edible plant species. The current paper discussed the plant species, containing seeds used as grain as wild grain, and the plant species whose underground parts of bulbs, roots, and rhizomes served as food substitutes (Table 2).

**Table 2:** Ethnobotanical inventory of wild grain and some plants which as food substitutes in FPM

Scientific name	Local name	Parts used	Purposes	Usage
*Agriophyllum squarrosum* (L.) Moq	čűrxil, sűlxir,siyorkűl	Seeds	Grain	Rice, flour
*Corispermum mongolicum* Iljin	xoron xamxag, xaraldai xamxag	Seeds	Grain	Parched flour
*Kalidium gracile* Fenzl	sir_a bűdűrgan_a, sir_a modo	Seeds	Grain	Parched flour
*Leymus racemosus* (Lam.) Tzvel	suli, xar_a suli, čagan suli	Seeds	Grain	Rice, parched flour
*Lilium martagon* L	sir_a tűmis	Bulbs	Refer as food substitutes	–
*Lilium pumilum* DC	čagan tűmis, saran_a	Bulbs	Refer as food substitutes	Boiled in milk; fresh bulbs are eaten raw; stored in cream or oil
*Paeonia anomala* L	yagan čen_e, čen_e	Roots	Refer as food substitutes	Boil the roots or made into like oat flour
*Paeonia lactiflora* Pall	čagan čen_e, čen_e, mandaraw_a čeneg	Roots	Refer as food substitutes	Boil the roots or made into like oat flour
*Polygonum viviparum* L	mexer	Rhizomes	Refer as food substitutes	Flour
*Potentilla anserina* L	sičigene	Roots	Refer as food substitutes	–
*Psammochloa villosa* (Trin.) Bor	suli, xar_a suli	Seeds	Grain	Flour, rice
*Rheum nanum* Siev. ex Pall	badǰűn_a	Roots	Refer as food substitutes	Flour

*Local names are spelled with the Mongolian orthography dictionary (revised edition) (Surgelet editors 2011)

Many grain types and some plants fetched from the wild were collected to make up a significant amount of food proportions. The use of food substitutive plants might be subject to the shortage of grain supply in Mongolia. Thus, the nomadic lifestyle in Mongolian regions could not guarantee a consistent grain supply since the nomadic Mongolians had not attained agricultural skills and experience insufficient dietary supplementation of starch. However, they tackled this by collecting wild grain and some plants as food substitutes from viable plants to meet the dietary starch demand.

Among wild grain and plants with some plants as food substitutes, the intake method of bulbs of *Lilium pumilum* was carefully conjugated with the preparation of traditional local dairy products. The collection method for *Polygonum viviparum* (common name: alpine bistort) rhizomes involved taking mice's hoard food. Local Mongolians referred to a gathering of rhizomes hoard of mice as "opening the alpine bistort palace (mine)"(Badamkhatan, 1987). It demonstrated the special ecological relationship among people, plants, and animals.

Mongolians have a long history of eating bulbs of *Lilium pumilum*, and it can be traced back to the end of the twelfth century (Khasbagan, 1996) when Genghis Khan was a child. The Mongolian residents of Arhorchin Banner, Xilingol League, Ordos plateau, and Ejina Banner of China are still collect the seeds of *Agriophyllum squarrosum* as a source of wild grain (Khasbagan et al.,^2000^, ^2005^, ^2011^; Man et al., 2007). *Agriophyllum squarrosum* was probably the most common wild plant as grain for Mongolians. Mongolians in the Ordos plateau also collect seeds of *Psammochloa villosa* as grain supplements (Man et al., 2007). Genus *Corispermum* and *Kalidium* are important wild grain plants used by Mongolians. FPM has shown that Mongolians in Mongolia had been using the seeds of *Corispermum mongolicum* as grain, whereas another species of Corispermum dilutum (Kitag.) C. P. Tsien & C. G. Ma, *Corispermum declinatum* Iljin gained popularity in Inner Mongolia (Khasbagan et al., 2000;Man et al., 2007). Mongolians in Mongolia used the seeds of *Kalidium gracile* as grain. Still, the Mongolians in Inner Mongolia preferred another species of *Kalidium foliatum* (Pall.) Moq.(Khasbagan et al., 2005). The Mongolians from Mongolia and Ejina Banner of Inner Mongolia shared the same practice of using flesh roots of *Rheum nanum* as food substitutes (Khasbagan et al., 2005). The bulbs of *Lilium martagon* and *Lilium pumilum* had been used as food substitutes in Mongolia. Still, Inner Mongolians select the bulbs of *Lilium pumilum* and *Lilium concolor* Salisb. var. *pulchellum* (Fischer) Regel as fruit or snacks and consumed it raw (Khasbagan et al.,). However, the young plant of *Lilium pumilum* and *Potentilla anserina* were consumed as vegetable by the Mongolians in Daqinggou of Inner Mongolia, China (Sachula et al., 2020).

#### Wild vegetables

The original records of wild vegetables in FPM were translated as follows:

- *Allium altaicum* Pall.: The locals collected the juicy and sweet bulbs (weighing up to 100 g) for food. The green leaves cannot be used for food because of their extensive fiber content. According to the description (1881–1883) of G. N. Potanin, *Allium altaicum* was collected in Khangai district and exported to Urgoo (Уpгy). Before the Chinese revolution, commercial companies exported thousands of kilograms of bulbs from Uliastay to China.
- *Allium leucocephalum* Turcz.: Local people use it as food.
- *Allium lineare* L.: Local people use it as food.
- *Allium ramosum* L. [*Allium odorum* L.]: Local people use it as food.
- *Allium senescens* L.: It is considered a very resourceful plant by the locals. For example, the bulbs, the lower part of the stems, and the tender leaves could all used for food. The harvested fresh plant could be purchased and stored for later use in winter. It is required to processing through fermentation, drying, or crushing before storage. Sometimes it could be mixed with goat cheese (aaruul) and baked into pancakes.
- *Allium victorialis* L.: The tender leaves can be consumed in fresh or salted state. It is sold in large quantities in the local markets of Ulaanbaatar and Altanbulag.
- *Cynanchum thesioides* (Freyn) K. Schum.[*Antitoxicum sibiricum* (L.) Pobed.]: The locals used its young fruit for making pickles.
- *Pugionium dolabratum* Maxim.[*Pugionium cristatum* Kom.]: The herdsmen salted the young plants of *Pugionium dolabratum* and consumed.
- *Ulmus pumila* L.: The local people cooked the immature fruit in salted water.

Wild vegetables were sourced from uncultivated plants such as part of a young plant, tender leaves, young fruits, the lower part of stems, and bulbs, comprising nine wild plant species that account for 25.71% of all edible plant species (Table 3). The nomadic Mongolians had not developed vegetable planting techniques in the past. Thus, they relied on the simple collective method of uncultivated vegetables from the wild.

**Table 3:** Ethnobotanical inventory of wild vegetables in FPM

Scientific name	Local name	Parts used
*Allium altaicum* Pall	songgin, ǰumagil, sogono, savimsag	Bulbs
*Allium leucocephalum* Turcz	gogod	Not described in FPM; could be tender leaves
*Allium lineare* L	gogod	Not described in FPM; could be tender leaves
*Allium ramosum* L	gogod	Not described in FPM; could be tenderleaves
*Allium senescens* L	manggir	Bulbs, the lower part of stems, tender leaves
*Allium victorialis* L	xaliyar	Tender leaves
*Cynanchum thesioides* (Freyn) K. Schum	teinegeni xűx	Young fruits
*Pugionium dolabratum* Maxim	ǰerlig lobang	Young plant
*Ulmus pumila* L	xayilyas	Young fruits

Among the wild vegetables, six species were reported as Allium. Edible parts of *Allium senescens* consisted of bulbs, the lower part of the stems, and the tender leaves. Its storage method was fully integrated with the characteristics of living tradition among nomadic people. Yunatov provided the trade history of *Allium altaicum* in the 1880s according to the description (1881–1883) of G. N. Potanin (Potanin, 1881–1883).

Mongolians have a long history of consuming *Allium ramosum*, *Allium senescens,* and *Allium victorialis* for diet compositions. Such dietary application can be traced back to the twelfth century (Volkov & Rachkovskaya, 2009). It is reported that nine of *Allium* species were used as vegetables, non-staple food, and seasonings by the Inner Mongolians (Khasbagan and Pei, 1999). Among them, *Allium ramosum* had been very special in terms of use (Khasbagan et al., 1999). The Mongolians in Arhorchin Banner also used *Allium ramosum*, *Allium senescens*, *Allium victorialis*, *Cynanchum thesioides*, *Ulmus pumila* as wild vegetables (Khasbagan et al.,^2000^). In addition to using *Cynanchum thesioides* and *Ulmus pumila* as vegetables, Mongolians in Arhorchin Banner also included young fruits of the above species as fruits in their dietary structure (Khasbagan et al., 1995). Tender leaves and the inflorescence of *Allium ramosum* and *Allium senescens*, and young fruits of *Cynanchum thesioides* were consumed as vegetables by the Mongolians in Daqinggou, Inner Mongolia, China (Sachula et al., 2020). The Mongolians in Alashan Left Banner of Alashan League used young fruits of *Cynanchum thesioides* as edible fruits (Khasbagan et al., 2000). In Xilingol League, Mongolians also used *Allium ramosum*, *Allium senescens*, *Cynanchum thesioides* as wild vegetables (Khasbagan et al., 2011). The Mongolians in the Ordos plateau also used *Cynanchum thesioides*, *Pugionium dolabratum*, *Ulmus pumila* as wild vegetables. In addition to *Pugionium dolabratum*, *Pugionium cornutum* (L.) Gaertn was also served as vegetable (Man et al., 2007). Instead of using *Pugionium dolabratum*, the Mongolians in Ejina Banner of Alashan League used another species of *Pugionium cornutum* (L.) Gaertn. as vegetables (Khasbagan et al. 2005).

#### Wild fruits

The original records of wild vegetables in FPM were translated as follows:

- *Fragaria orientalis* Losinsk.: The fruit is bright red and edible.
- *Malus baccata* (L.) Borkh.[*Malus pallasiana* Juz.]: Small spherical fruits (up to 1 cm in diameter) serve as food for local people.
- *Nitraria sibirica* Pall.: Local people use its fruit for food.

There are only three wild fruits species, accounting for 8.57% of all edible plant species (Table 4).

**Table 4:** Ethnobotanical inventory of wild fruits listed in FPM

Scientific name	Local name	Parts used
*Fragaria orientalis* Losinsk	gűǰelǰegen_e	Fruits
*Malus baccata* (L.) Borkh	űril, űlir	Fruits
*Nitraria sibirica* Pall	tobčog, sűndűl, xaymag	Fruits

It is reported that *Malus baccata* have been consumed as a fruit by Mongolians for a long time. *Malus baccata* was used as wild fruit in Arhorchin Banner and Xilingol League (Khasbagan et al., 2000, 2011, 1995). Also, there are records of using *Nitraria sibirica* as wild fruit in the Ordos plateau, Ejina Banner, and the Alshan Left Banner of Alashan League. Apart from that, *Nitraria sibiric*a, *Nitraria tangutorum* Bobr. were also used; furthermore, *Nitraria roborowskii* Kom. were also consumed as wild fruits (Khasbagan et al., 2005; Man et al., 2007; Soyolt et al., 1999).

#### Tea substitutes

The original records of tea substitutes in FPM were translated as follows:

- *Bergenia crassifolia* (L.) Fritsch: Used as tea substitutes.
- *Clematis hexapetala* Pall.: The locals widely use it as tea substitutes.
- *Geranium pseudosibiricum* J. Mayer: Local people widely use it as tea substitutes.
- *Paeonia anomala* L.: People use it in the same way as the previous species (The author note: the earlier species in FPM was *Paeonia lactiflora*).
- *Potentilla fruticosa* L.: Local people used it as tea substitutes since ancient times.
- *Rosa acicularis* Lindl.: Used as tea substitutes.
- *Sanguisorba officinalis* L.: Leaves and roots are used as tea substitutes.

Tea substitutes are sourced from leaves, roots, and the aboveground parts of possibly eight wild plants species, accounting for 22.85% of all edible plant species (Table 5). Drinking milk tea had become one of the characteristics of the Mongolian diet and culture since the rise of the Mongolian Empire and the Mongolian rule in the Yuan dynasty in the twelfth century (Cai, 1994). Mongolians have been drinking brick tea (a type of imported, compressed black tea, green tea or pu-erh leaves) for centuries. The habit of drinking tea meets the dietary nutritional supplements that lack in the main meal compositions. It can be seen as the direct driving force of choosing and using tea substitutes from Mongolians' local wild plants. Since the brick tea is usually obtained by trade, the tea substitutes sourced from the wild can relieve the shortage of tea when the brick tea may be intermediately unavailable for purchase in a period.

**Table 5:** Ethnobotanical inventory of tea substitutes in FPM

Scientific name	Local name	Parts used
*Bergenia crassifolia* (L.)Fritsch	badan	Not described in FPM; could be aboveground parts
*Clematis hexapetala* Pall	ǰogdir	Not described in FPM; could be aboveground parts
*Geranium pseudosibiricum* J. Mayer	Miyagmasanǰa, dűgűr xorlo	Not described in FPM; could be aboveground parts
*Paeonia anomala* L	yagan čen_e, čen_e	Roots, follicle
*Paeonia lactiflora* Pall	čagan čen_e, čen_e, mandaraw_a čeneg	Roots, follicle
*Potentilla fruticosa* L	ṧűgűr, borolǰigan ṧűgűr, buryagűl, dalan xalisu	Not described in FPM; could be aboveground parts
*Rosa acicularis* Lindl	noxayiu xűṧű, űlan xalaxay	Not described in FPM; could be leaves
*Sanguisorba officinalis* L	Siyod űbs, sűd	Leaves and roots

Plant roots such as Sanguisorba officinalis were reported as a vital food source whose dietary use could be traced back to the end of the twelfth century (Khasbagan, 1996). *Clematis hexapetala* (stems & leaves), *Paeonia lactiflora* (Follicle without seed), and *Sanguisorba officinalis* (roots & stems) were reported as traditional tea substitutes of the Mongolians in Inner Mongolia (Khasbagan, 1990). The Mongolians in Arhorchin Banner have been using *Clematis hexapetala*(stems and leaves), *Paeonia lactiflora* (follicle, and Sanguisorba officinalis (roots, stems)for viable tea substitutes. However, *Potentilla fruticosa* and *Rosa acicularis* were reported with lesser use, and an alternative species of *Potentilla chinensis* Ser. (aboveground parts) and *Rosa davurica* Pall. (leaves, flowers, fruits) might have been used as tea substitutes (Khasbagan et al., 1996, 2000). Also, *Sanguisorba officinalis* L. (roots stems) were reported as preferred tea substitutes by Mongolians in Xilingol League. However, they tended not to use *Potentilla fruticosa,* but, instead, an alternative species of *Potentilla anserina* L. (leaves) (Khasbagan et al., 2011).

#### Wild seasonings

The original records of wild seasonings in FPM were translated as follows:

- *Allium lineare* L.: Seasoning in soups and meats when fresh and dry.
- *Allium mongolicum* Regel: Gobi herders especially like to use fresh or dried (crushed) *Allium mongolicum* as meat seasoning. In this regard, herders prefer *Allium mongolicum* to *Allium polyrhizum*.
- *Allium polyrhizum* Turcz. ex Regel: Sometimes, people use it for seasoning food.
- *Nepeta annua* Pall.[*Schizonepeta annua* (Pall.) Schischk.]: Seeds are used as flavoring for meat.
- *Polygonum viviparum* L.: Cooked as a seasoning for meat.
- *Saposhnikovia divaricata* (Turcz.) Schischk: The local people use the seeds as seasoning of meat.
- *Sisymbrium heteromallum* C. A. Mey.: In the Gobi Altai region, its seeds are used as pungent condiments in food.

Seasonings from wild sources consist of plants from seeds, rhizomes, and tender leaves (possibly), comprising seven species of wild plants, accounting for 20.00% of all edible plant species (Table 6).

**Table 6:** Ethnobotanical inventory of wild seasonings in FPM

Scientific name	Local name	Parts used
*Allium lineare* L	gogod	Not described in FPM; could be tender leaves
*Allium mongolicum* Regel	xűműli	Not described in FPM; could be tender leaves
*Allium polyrhizum* Turcz. ex Regel	tan	Not described in FPM; could be tender leaves
*Nepeta annua* Pall	bibiling, bandűi	Seeds
*Polygonum viviparum* L	mexer	Rhizomes
*Saposhnikovia divaricata* (Turcz.) Schischk	gonid	Seeds
*Sisymbrium heteromallum* C.A.Mey	borbot	Seeds

The tender leaves of *Allium polyrhizum* were served as vegetables or inflorescence as seasonings by Mongolians in Arhorchin Banner (Khasbagan et al. 2000). However, in Xilingol League, Mongolians used tender leaves of *Allium mongolicum* and *Allium polyrhizum* as vegetables and herbs (Khasbagan et al., 2011). In contrast, Mongolians from Ordos plateau used the leaves of Allium mongolicum as vegetables and seasonings and the inflorescence of *Allium polyrhizum* for seasonings herbs ( Man et al., 2007). Ejina Banner reported examples to use tender leaves and inflorescence of *Allium mongolicum* as vegetables and inflorescence of *Allium polyrhizum* as culinary seasonings (Khasbagan et al., 2005). Meat and milk are the main elements in the traditional diet structure of Mongol herders. The consumption of *Lilium pumilum*, *Allium senescens*, *A. lineare*, *Nepeta annua*, *Polygonum viviparum,* and *Saposhnikovia divaricata* was tightly integrated with the meat and dairy intake of the locals.

There are many differences in the selection and utilization of wild plants among Mongolian banners in different regions. In the wild grain categories and some plants, there is a significant level of similarities in the usage of food substitutes in both Inner Mongolia and the Republic of Mongolia. For example, *Agriophyllum squarrosum, Psammochloa villosa, Rheum nanum, and Lilium pumilum*, were commonly used by Mongolians in both countries. Mongolians seemed to typically use Corispermum, Kalidium, and Lilium genera in both countries, but the exact species' selection proved different. In the categories of wild vegetables, Mongolians in both countries consume *Allium ramosum*, Allium senescens, *Allium victorialis*, *Cynanchum thesioides*, *Pugionium dolabratum Ulmus pumila* for vegetables. Still, sometimes a slight difference in intake methods exists. *Corispermum*, *Kalidium,* and *Lilium* were taken as the equivalent dietary composition of the Mongolians in both countries; however, there were differences in the selected species. In the wild fruit categories, *Malus baccata* were reported in typical consumption in both countries. *Nitraria tangutorum* and *Nitraria roborowskii* were seen as wild fruits in Inner Mongolia, Clematis hexapetala, Paeonia lactiflora *Sanguisorba officinalis* were popular tea substitutes in both Inner Mongolia and Mongolia. Mongolians in both countries regularly consumed *Potentilla* and *Rosa* but different in the selected species. Despite that, Mongolians in both countries used Allium mongolicum and Allium polyrrhizum as seasonings ingredients in the wild seasoning categories.

It has been seen as early as in the twelfth century when *Lilium pumilum*, *Allium ramosum*, *Allium senescens*, *Allium victorialis*, *Malus baccata*, *Sanguisorba officinalis* have been collected and used for food by Mongolians. It demonstrates that the knowledge of these plants' application by Mongolians has a long history, from the Genghis Khan era to the present day, representing significant cultural and historical value.

## Conclusions

Although Yunatov was not an ethnobotanist himself, he faithfully recorded the data obtained from interviews and surveys about the Mongolians in Mongolia regarding the collection and consumption of local wild plants during 1940–1951. He devoted his research mission to collecting valuable first-hand knowledge on forage grass, natural feed of livestock, and wild plants for human consumption. Despite incomplete information on some plants' edible parts and eating methods, it has achieved a remarkable research outcome considering the content was completed more than 70 years ago. Thus, FPM demonstrated a valuable reference of historical and ethnobotanical information. Even with present-day standards, such research will still be challenging, primarily to obtain interviews and first-hand knowledge with correct interpretations to such extent and richness. However, further research is granted for field research and potential value of wild edibles in current day food nutrition.

## Data Availability

We have already included all data in this manuscript.

## Abbreviations

FPM: Fodder Plants of Pastures and Hayfields of the People’s Republic of Mongolia

## References

[CR1] Aryal KP, Poudel S, Chaudhary RP, Chettri N, Chaudhary P, Ning W (2018). Diversity and use of wild and non-cultivated edible plants in the Western Himalaya. Journal of Ethnobiology and Ethnomedicine.

[CR32] Badamkhatan, S. (1987). *G.N.Potanin travel in Mongolia.* National Press (in Mongolian).

[CR2] Bhatia H, Sharma YP, Manhas RK, Kumar K (2018). Traditionally used wild edible plants of district Udhampur, J&K. India. Journal of Ethnobiology and Ethnomedicine.

[CR3] Cai ZC (1994). Talking about the Mongolian tea culture. The North of Cultural Relics.

[CR4] Engler A (1930). Hydrangea in engler and prantl. The Natural Plant Families.

[CR5] Enhebayar, Soyolt, Khasbagan (2002). Nutritional components in fruits of *Ribes pulchellum* used as edible wild fruits by the Arhorchin Mongolians. Acta Nutrimenta Sinica.

[CR6] Gilbaa K (2019). Textual research on the Mongol names of gramineous forage in the book advice to the people on how to manage animal husbandry. Journal of Inner Mongolia Normal University Natural Science Edition in Mongolian.

[CR7] Huai KHY, Pei SJ (2000). Wild plants in the diet of Arhorchin Mongol herdsmen in Inner Mongolia. Economic Botany.

[CR8] Ju Y, Zhuo JX, Liu B, Long CL (2013). Eating from the wild: diversity of wild edible plants used by Tibetans in Shangri-la region, Yunnan. China. Journal of Ethnobiology and Ethnomedicine.

[CR9] Kang Y, Luczaj L, Kang J, Wang F, Hou JJ, Guo QP (2014). Wild food plants used by the Tibetans of Gongba Valley (Zhouqu county, Gansu, China). Journal of Ethnobiology and Ethnomedicine.

[CR10] Karamysheva ZV (2009). AA.Yunutov and his work in Mongolia. Vegetation of Russia.

[CR11] Khasbagan (1996). The study of wild edible plants in *the** secret history** of the Mongolians*. Journal of Arid Land Resources and Environment.

[CR12] Khasbagan N, Stuart K (1999). Ethnobotanical overview of gogd (Allium ramasum L.): A traditional edible wild plant used by Inner Mongolians. Journal of Ethnobiology.

[CR13] Khasbagan, Pei, S. J. (1999). Ethnobotanical study on *Allium* L. in grassland of Inner Mongolia. *Chinese Journal of Grassland, 5*, 42–47. (in Chinese).

[CR14] Khasbagan (1990). A preliminary study on plants used as traditional Mongolian tea in Inner Mongolia. Acta Botanica Yunnanica.

[CR15] Khasbagan (1996). Ethnobotanical study of the Mongolians utilizing the Chinese Ephedra (*Ephedra sinica* Stapf). Journal of Inner Mongolia Normal University (natural Science Edition in Chinese).

[CR16] Khasbagan (2007). *Ephedra sinica* Stapf (Ephedraceae): The fleshy bracts of seed cones used in Mongolian food and its nutritional components. Economic Botany.

[CR17] Khasbagan, Geng XH, Orgil JF, Chen S (2007). Nutritional contents of the fruits of Ribes diacanthum Pall and its evaluation of edible value. Journal of Inner Mongolia Normal University Natural Science Edition in Chinese.

[CR18] Khasbagan, Man L, Enhebayar G, Hu W (2005). Traditional usage of wild plants for food by the ejina Mongolians and its exploitation and ethnoecological significance. Journal of Inner Mongolia Normal University Natural Science Edition in Chinese.

[CR19] Khasbagan., Enhebayar., & Imzab. (1995). Ethnobotanicl study on wild edible fruits of the Mongolians in Arhorchin Banner, Inner Mongolia. *Journal of Inner Mongolia Normal University(Natural Science Edition), 23*(1), 60–63 (in Chinese).

[CR20] Khasbagan., Soyolt., & Imzab. (1996). Ethnobotanical study on tea substitute plants of the Mongolians in Arhorchin Banner, Inner Mongolia, China. *Journal of Inner Mongolia Normal University(Natural Science Edition), 24*(4), 62–65 (in Chinese).

[CR21] Khasbagan., Yeruhan., & Zhao, H. (2011). Study on traditional knowledge of wild edible plants used by the Mongolians in Xilingol typical steppe area. *Plant Diversity and Resources, 33*(2), 239–246. 10.3724/SP.J.1143.2011.10158 (in Chinese).

[CR22] Ligaa, U., Davaasuren, B., & Ninjil, N. (2006). *Medicinal plants of Mongolia used in western and eastern medicine*. Ulaanbaatar JCK Press (in Mongolian Cyrillic).

[CR23] Łuczaj Ł, Köhler P, Pirożnikow E, Graniszewska M, Pieroni A, Gervasi T (2013). Wild edible plants of Belarus: From Rostafiński’s questionnaire of 1883 to the present. Journal of Ethnobiology and Ethnomedicine.

[CR24] Lulekal E, Asfaw Z, Kelbessa E, Van Damme P (2011). Wild edible plants in Ethiopia: A review on their potential to combat food insecurity. Afrika Focus..

[CR25] Magsar U, Baasansuren E, Tovuudorj ME, Shijirbaatar O, Chinbaatar Z, Lkhagvadorj K (2018). Medicinal plant diversity in the southern and eastern Gobi Desert region. Mongolia. Journal of Ecology and Environment.

[CR26] Mandakh U, Battseren M, Ganbat D, Ayanga T, Adiya Z, Borjigidai A (2020). Folk nomenclature of plants in Cistanche deserticola-associated community in South Gobi Mongolia. Plant Diversity..

[CR27] Manliang ZXS, Khasbagan E (2007). Study on the Mongolian traditional knowledge of wild edible plants in Ordos Plateau. Acta Botanica Yunnanica.

[CR28] Martin GJ (1995). Ethnobotany: A methods manual.

[CR30] Pei, S. J. (1988). Ethnobotany and exploitation of plant resources. *Acta Botanica Yunnanica,* [supplement I], 135–144 (in Chinese).

[CR31] Potanin, G. N. (1881–1883). *Essays on Northwest Mongolia, 1*(4). St. Petersburg Folkzos Lozov Printing House (in Russian).

[CR33] Sachula G, Zhang Y-Y, Zhao H, Khasbagan K (2020). Wild edible plants collected and consumed by the locals in Daqinggou, Inner Mongolia, China. Journal of Ethnobiology and Ethnomedicine.

[CR34] Sambo J (1945). Advice to the people on how to manage animal husbandry.

[CR35] Soyolt., Khasbagan., Hong, Y., & Mei, L. J. (1999). Ethnobotanical study on wild edible fruits of the Mongol herdsmen in Alshan Leage, Inner Mongolia. *Journal of Inner Mongolia Normal University (Natural Science Edition), 28*(4). 10.3969/j.issn.1001-8735.1999.04.017 (in Chinese).

[CR36] Surgelet (2011). Mongolian orthography dictionary.

[CR29] Online document The Herbarium of Vascular Plants of the Komarov Botanical Institute (2006, March 25) Collections of A. A. Junatov from Central Asia (DB /OL)*, The Herbarium of Vascular Plants of the Komarov Botanical Institute (LE)*. Retrieved January 7, 2020, from http://www.mobot.org/MOBOT/Research/LEguide/collections/19/index.html.

[CR37] Urgamal M, Gundegmaa V, Baasanmunkh Sh, Oyuntsetseg B, Darikhand D, Munkh-Erdene T (2019). Additions to the vascular flora of Mongolia-IV. Proceedings of the Mongolian Academy of Sciences.

[CR38] Urgamal M, Oyuntsetseg B (2017). Atlas of the endemic vascular plants of Mongolia.

[CR39] Volkov EA, Rachkovskaya EI (2009). A contribution by A. A Yunatov to the knowledge of the vegetation of XinJiang. Vegetation of Russia.

[CR40] Wujisguleng W, Khasbagan K (2010). An integrated assessment of wild vegetable resources in Inner Mongolian Autonomous Region. China Journal of Ethnobiology and Ethnomedicine.

[CR41] Wurchaih W, Khasbagan K (2017). A preliminary investigation of wild plants used by the Mongolians in Bairin Left Banner, Inner Mongolia, China: A case study in Chaganhad. Nature of Inner Asia.

[CR42] Yang J, Chen W-Y, Fua Y, Yanga T, Luo X-D, Wang Y-H (2020). Medicinal and edible plants used by the Lhoba people in Medog County, Tibet. China. Journal of Ethnopharmacology.

[CR43] Yunatov AA (1954). Fodder plants of pastures and hayfields of the People’s Republic of Mongolia.

[CR44] Yunatov AA, Huang ZH, Ma YQ, Wang JW (1958). Fodder plants of pastures and hayfields of the People’s Republic of Mongolia.

[CR45] Yunatov AA, Rdenejev GE (1968). Fodder plants of pastures and hayfields of the People’s Republic of Mongolia.

[CR46] Zhang, Y. Y., Khasbagan. (2016). Ethnobotanical value of the book Range management written by Prof Wang Dong. *Journal of Inner Mongolia Normal University (Natural Science Edition), 45*(6), 840–848. 10.3969/j.issn.1001-8735.2016.06.023 (in Chinese).

[CR47] Zhiming L, Huinuan L, Long G, Jingwen G, ChiMeng TZ (2016). Herba Cistanche (Rou Cong-Rong): One of the best pharmaceutical gifts of traditional Chinese medicine. Front Frontiers in Pharmacology.

